# Pathophysiology and Evolving Treatment Options of Septic Arthritis: A Narrative Review

**DOI:** 10.7759/cureus.65883

**Published:** 2024-07-31

**Authors:** Alan D Kaye, Driskell Greene, Alana V Alvarez-Amado, Horace L Townsend, Michael Forte, Megan Vasterling, Jon D Hirsch, Jeffrey Howard, Shahab Ahmadzadeh, Olga Willett, Adam M Kaye, Sahar Shekoohi, Giustino Varrassi

**Affiliations:** 1 Department of Anesthesiology, Louisiana State University Health Sciences Center, Shreveport, USA; 2 Department of Medicine, Louisiana State University Health Sciences Center, Shreveport, USA; 3 School of Medicine, American University of the Caribbean, Cupecoy, SXM; 4 School of Medicine, Louisiana State University Health Sciences Center New Orleans, New Orleans, USA; 5 Department of Pharmacy Practice, Thomas J. Long School of Pharmacy and Health Sciences, University of the Pacific, Stockton, USA; 6 Department of Pain Medicine, Paolo Procacci Foundation, Rome, ITA

**Keywords:** antibiotic, articular involvement, streptococcus, staphylococcus, pyogenic arthritis, septic arthritis

## Abstract

Pyogenic (septic) arthritis is a severe joint infection characterized by the invasion of microorganisms into the synovium, causing inflammation and joint destruction. This review article provides a comprehensive overview of pyogenic arthritis, focusing on etiology, pathogenesis, clinical manifestations, diagnosis, and management strategies. This review explores routes of microbial entry into joints, emphasizing the importance of prompt identification and treatment to prevent irreversible joint damage. Clinical manifestations, such as joint pain, swelling, and limited range of motion, are discussed, along with the challenges in differentiating pyogenic arthritis from other joint disorders. Diagnostic approaches, including joint aspiration and imaging modalities, are critically examined for accuracy in confirming diagnosis. This review also addresses the significance of early intervention through antimicrobial therapy and joint drainage, highlighting the role of multidisciplinary collaboration in optimizing patient outcomes. In summary, the present investigation underscores the complexities of pyogenic arthritis and the need for a comprehensive understanding of pathophysiology for timely and effective management to improve patient prognosis and quality of life.

## Introduction and background

Pyogenic arthritis (also referred to as septic arthritis) is an infection of one or multiple joints, which illustrates the tenuous relationship between the synovial environment and its constant need to function free from microbial pathogens. Various microbial pathogens' invasion of the joint space causes many clinical manifestations and challenges that require prompt and effective management [1]. A fundamental understanding of pyogenic arthritis remains crucial for healthcare providers and is essential to its management. Pyogenic arthritis is a multifaceted disease requiring multiple considerations regarding etiology, pathophysiology, clinical management, and differential diagnoses. A wide variety of microbial organisms lead to the development of pyogenic arthritis [2]. The primary culprits are bacteria, commonly *Staphylococcus aureus*, different *Streptococcus *species, and other culprits, including Gram-negative bacilli 2.

Various causative pathogens are essential to establishing a prompt diagnosis while crafting an effective treatment plan. Related to the wide variety of clinical manifestations and somewhat nonspecific presenting symptoms, it may be challenging to recognize pyogenic arthritis [3], and the clinician needs to identify potential differential diagnoses as well. Articular involvement is common to most other diagnoses, such as avascular necrosis, osteomyelitis, and other forms of arthritis. Once a diagnosis is established and confirmed, an effective management plan is crucial for ensuring positive patient outcomes [4]. Various causative organisms, different treatment methods, and both the articular and systemic manifestations are essential considerations when crafting a safe and effective course of treatment. Most treatment plans combine treatments, including antimicrobial therapy, joint drainage, and culture via arthrocentesis and supportive care [4]. Providers must be vigilant in identifying and managing this disease, and this article will discuss identification, management, and differential diagnoses for pyogenic arthritis, among other relevant topics.

## Review

Etiology, epidemiology, and diagnosis

Pyogenic Arthritis

Pyogenic arthritis mainly presents clinically as an articular infection. The most commonly involved joints include the knee, hip, shoulder, and elbow [5]. A common mechanism of infection is via hematogenous spread to the vascular synovial membrane, and once infected, the cartilaginous matrix is rapidly destroyed. Bacterial inoculation of the synovial membrane can arise from penetrating trauma to the joint or from contiguous spread related to osteomyelitis or skin infection [6]. Expression of host-derived protein products meant to promote joint healing can inadvertently further promote bacterial colonization. Furthermore, low fluid within the enclosed joint space can further encourage bacterial infection [7]. The most implicated organisms in pyogenic arthritis include *S. aureus*, *Streptococcus pneumoniae*, and Gram-negative bacteria.

Most Common Etiological Agent

*S. aureus* is the most common organism responsible for pyogenic arthritis in the United States [6]. Reports of methicillin-resistant *S. aureus* infections have increased due to the continued development of antibiotic resistance, especially in cases with chronic medical conditions and IV drug users [7,8]. *S. pneumoniae* joint infection is seen more commonly in the elderly with pneumococcal bacteremia and potentially those with a history of *S. pneumoniae* vaccination. *Neisseria gonorrhoeae* is an agent for pyogenic arthritis that accounts for 0.6% to 1.2% of cases in North America [9] and is common in sexually active persons who engage in risky behaviors [9]. Although uncommon, *N. gonorrhoeae* joint infection may also present in individuals with a negative sexual history [9]. *Pseudomonas aeruginosa *is often seen in immunocompromised cases with chronic renal failure or those undergoing hemodialysis. Patients on hemodialysis have a high incidence of skin infections due to repeated skin trauma associated with administering dialysis [10]. Other less commonly implicated organisms include *Pasteurella multocida *and *Eikenella corrodens*. However, these infections present with a history of human and animal bites. Occasionally, the causative agent of joint infection may remain unidentified. This is likely related to three factors: (a) prior antibiotic use before tissue sampling, (b) organism absence in the joint with an abundance of inflammatory mediators, and (c) inappropriate culturing conditions.

Organisms implicated in pyogenic arthritis, virulence factors, and immunological activation

*S. aureus *and *S. pneumoniae* share virulence factors integral to joint infection. Such virulence factors include fibronectin-binding proteins (FNBPs) [11]. FNBP gene knockout models revealed that FNBP is required for full virulence, as exhibited by weight loss, organ abscesses, and joint infections [12]. A separate study of transposon gene FNBP knockout models revealed minimal-to-no *S. aureus* aggregation in synovial joint fluid membranes [13]. Similarly, gene knockout of FNBPs pavA in *S. pneumoniae* revealed a significant decrease in cell infection [14]. These findings indicate the role FNBPs play in the aggregation of *S. aureus *and *S. pneumoniae* in synovial fluid and the pathogenesis of pyogenic arthritis.

*S. pneumoniae *and *N. gonorrhoea* express surface proteins that promote cell infectivity. Immunofluorescence tagging of *S. pneumoniae *RrgA pili protein revealed a transient association between the bacterium and fibronectin-host protein [15]. A separate study showed that bacteria deficient in RrgA displayed decreased fibronectin adherence compared to the wild-type sample, while RrgA overexpression enhanced adherence [16]. Furthermore, the density of bacteria colonizing the upper respiratory tract of mice with RrgA-negative pneumococci was significantly less than the wild type, exhibiting decreased bacteremia and joint infection susceptibility [16]. Similarly, *N. gonorrhoea's* outer membrane PorB1A proteins are integral to disseminating gonococcal infections [17]. Specifically, the arginine PorB1A residue is a highly conserved sequence, and changes have led to the loss of the invasive phenotype [17]. Interestingly, low phosphate conditions mediate PorB1A-fibronectin binding, which means the low phosphate environment found in circulation may promote PorB1A-receptor interaction and contiguous bacterial spread.

Joint bacterial infection stimulates the recruitment of immune cells and the production of pro-inflammatory cytokines. Murine synovial tissue samples of septic joints revealed a large influx of macrophages, neutrophils, tumor necrosis factors (TNF-α), and interferons (IFN-γ) [18]. Other findings have shown that *N. gonorrhoea* pili activate CD4+ T-cells and stimulate the secretion of anti-inflammatory cytokines [17]. These immunological mediators act on the joint and promote bone resorption by forming an infectious nidus [19]. Interestingly, anti-TNF-α modulators have been shown to improve the severity of joint disease. However, complete inhibition of TNF-α is a risk factor for systemic infection [20]. Besides pro-inflammatory responses, macrophages are required to remove dying neutrophils as their persistence can contribute to chronic inflammation and tissue destruction. It has been proposed that the host's inflammatory response against the pathogen results in collateral damage, increasing mortality rates, and joint destruction.

Risk factors associated with pyogenic arthritis

Several risk factors contribute to the pathogenesis of pyogenic arthritis. The regression-variable analysis identified positive associations between smoking, increasing age, rheumatoid arthritis, skin infections, and extensive joint involvement with pyogenic arthritis [21]. A common risk factor found throughout literature discussing pyogenic arthritis is the increased risk of infection. Therefore, diabetes and immunosuppressive medications predispose patients to pyogenic arthritis through bacteremia and hematogenous spread [21]. There have been reports of diabetic patients with uncommon joint involvement and bacterial infections that infect humans infrequently. For example, in acromioclavicular joint infections, 20% of reported cases were diabetics, and 25% were on immunosuppressive therapy [22]. Similarly, a diabetic patient was found to have *Actinomyces pyogenes* ankle infection, an organism typically found in cattle [23]. As diabetes is confirmed as an independent risk factor of mortality in pyogenic arthritis, early identification of these risk factors is imperative for effective management.

Patients with underlying joint disease (rheumatoid arthritis (RA), osteoarthritis (OA), prosthetic joints (PJs)) have increased susceptibility to developing pyogenic arthritis. This poor prognosis is due to diagnostic delays as symptoms are sometimes misdiagnosed due to pre-existing joint pathology. Proposed theories suggest that abnormal joint remodeling and neovascularization in OA and RA promote bacterial colonization of the subchondral bone and synovium [24]. Skin lesions are the most common source of joint infections in patients with underlying joint disease, often from lymph nodes draining rheumatoid nodules or ulcerated foot calluses [24]. Furthermore, immune system dysfunction seen in chronic diseases increases the risk of joint infection. For example, neutropenia is common in chronic RA or in patients taking immunosuppressive therapy, and reports indicate a 10-fold risk of developing joint infection in neutropenic patients [18]. These findings suggest the relevance of underlying joint disease and associated increased risk of joint infection.

Joint arthroplasty procedures are another common cause of joint infections. Reports show a 1%-2% increase in prosthetic joint infections in patients with a background of OA and up to 4.4% in patients with RA [25]. Newly implanted joints undergo environmental remodeling through fibrinogen and fibronectin coating [18], with these host proteins serving as potential bacterial adherence points. Polymethyl methacrylate (PMMA), a space-filling agent that holds the implant against the bone, has been shown to have inhibitory activity against leukocytes and complement proteins [26]. Additionally, the heat released by the cementing agent during the surgical procedure creates a non-vascularized area next to the cortical bone, promoting a new environment for bacterial growth away from immune defenses [18]. Clinicians' understanding of risk factors that promote joint infection is crucial for early pyogenic arthritis treatment.

Lyme arthritis is a common feature of *Borrelia burgdorferi* infection, a type of infectious arthritis common to the Northeastern United States. Patients report symptoms of low-grade fever, migratory joint pain, and large synovial effusions. Lyme arthritis synovial inflammation may persist despite antibiotic therapy, referred to as antibiotic-refractory arthritis [27]. Lyme arthritis can resemble pyogenic arthritis in adults or juvenile idiopathic arthritis in children, and serologic testing is required to confirm Lyme diagnosis while ruling out other causes. Moreover, elevated levels of synovial leukocytes in Lyme arthritis can suggest a diagnosis of pyogenic arthritis. Chronic polyarthritis is rare in Lyme disease, a distinguishing factor from RA. Furthermore, other inflammatory arthritis, like reactive or psoriatic arthritis, may arise months after a Lyme infection [28]. These overlapping features signify the importance of considering these possibilities in the differential diagnosis. Other immunocompromised states causing increased propensity of developing pyogenic arthritis include HIV+ patients and those with liver cirrhosis. Reports show an increased prevalence of joint infections in HIV patients, with *S. aureus* being the most common causative agent [18]. In HIV patients with chronic monoarticular arthritis, opportunistic pathogens such as *S. pneumoniae*, mycobacterial, and fungal species should be ruled out as possible contaminants [18]. Furthermore, HIV patients receiving immunosuppressive therapy have an increased risk of infection (i.e., TB reactivation in HIV patients receiving TNF-α therapy). Although not very common, several case reports described an association between pyogenic arthritis and liver cirrhosis. Liver cirrhosis leads to immune cell dysfunction, while excessive alcohol use is related to an increased risk of skin infections [21]. In addition, portal hypertension can promote bacteriemia and pyogenic arthritis [21]. Therefore, pyogenic arthritis diagnosis in febrile cirrhotic patients should be considered when other causes of infection have been ruled out. Other risk factors associated with pyogenic arthritis are shown in Table 1.

**Table 1:** Risk factors for pyogenic arthritis

Risk factors
Contiguous route: Skin infection close to the joint, cutaneous ulcerations
Direct route: Intra-articular injection, prosthetic joint placement
Systemic risk factors: Diabetes, immunosuppressive medication use, IV drug use, HIV infection, pre-existing joint disease (e.g., rheumatoid arthritis), sexual activity
Other: smoking history, Age >80 years

Diagnosis of pyogenic arthritis

Diagnosis of pyogenic arthritis requires arthrocentesis with synovial fluid analysis, laboratory, and imaging studies. The most useful diagnostic test is arthrocentesis with synovial fluid analysis from the affected joint. WBC counts greater than 50,000 cells/mm^3^ with neutrophilic predominance and left band shift show evidence of a bacterial infection [2]. Other laboratory tests to support infection include a complete blood count, an erythrocyte sedimentation rate, C-reactive protein, and blood cultures. Two sets of blood cultures should be assessed to rule out bacteremia. If *N. gonorrhoea* infection is suspected, one should obtain cultures from the cervix [2]. X-ray imaging may show widened joint spaces and edema-induced bulging of soft tissues. However, x-ray imaging may be normal and does not rule out joint infection [2]. MRI imaging is the most sensitive as it can reveal the extent of infectious changes in the joint and surrounding tissues.

Treatment and management

Management of pyogenic arthritis includes antimicrobial therapy and joint fluid aspiration. Fluid aspiration is accomplished using arthrotomy, arthroscopy, or regular needle aspiration [2]. An arthroscopy procedure utilizes a malleable cylinder with a video camera at the tip positioned within the joint through a minor incision [3]. A fluid aspiration should be considered for any patient with an inflamed joint without a proven diagnosis [29]. Once an artificial joint is infected, the treatment plan includes removing the joint and temporarily replacing it with a joint spacer, a device made with antibiotic cement. If a replacement joint cannot be removed, a provider may clean the joint and remove the battered tissue, but the artificial joint is kept in place. Preliminary intravenous antibiotics are given, followed by oral antibiotics over the following months, to avoid infection recurrence [3]. Intravenous antimicrobic treatment should commence quickly once joint fluid aspiration is complete and cultures are attained. Empirical antimicrobial treatment includes anti-staphylococcal treatment (nafcillin, oxacillin, or vancomycin for all ages and all risks) [2]. Nafcillin and oxacillin are beta-lactam antibiotics that inhibit penicillin-binding proteins (PBPs) by attaching to PBPs and impeding the final stage of cell wall bacterial synthesis. These antibiotics also stimulate cell lysis via autolysins. Common side effects include allergic reactions, nausea, vomiting, diarrhea, stomatitis, black or hairy tongue, and gastrointestinal irritation [30].

Vancomycin, a tricyclic glycopeptide antibiotic, inhibits the polymerization of peptidoglycans in bacterial cell walls. By adhering to D-alanyl D-alanine, vancomycin inhibits peptidoglycan synthase (a glucosyltransferase) and the P-phospholipid carrier, which prevents the synthesis of gram-positive bacteria and ultimately results in bacterial cell death. Common side effects include nephrotoxicity, hypotension, and hypersensitivity reactions such as anaphylaxis [31]. Intravenous antimicrobic treatment for nongonococcal pyogenic arthritis incorporates vancomycin targeting gram-positive microbiota, mainly when there is a suspicion of methicillin-resistant *Staphylococcus aureus* (MRSA). A third-generation cephalosporin, such as ceftriaxone, ceftazidime, or cefotaxime, is added for gram-negative coverage when the patient is immunocompromised, cultures return a negative gram stain, or the patient abuses intravenous drugs. Third-generation cephalosporins perform like beta-lactam antibiotics by inhibiting peptidoglycan synthesis within the bacteria cell wall. Common side effects include diarrhea, nausea, vomiting, abdominal pain, rash, and injection site reactions. Third-generation cephalosporin treatment increases the risk of superinfection and pseudomembranous colitis induced by *C. difficile* [32].

The treatment course includes two weeks of intravenous antimicrobic treatment and one to two weeks of oral antimicrobic therapy. *P. aeruginosa* infection may necessitate a more extended treatment course (four to six weeks). Gonococcal arthritis, caused by *N. gonorrhoeae*, is treated with intravenous ceftriaxone for 24-48 hours. When clinical improvement is seen, oral treatment is continued. If no improvement is seen clinically within five to six days, the joint is re-aspirated to rule out Lyme disease, the presence of fungi, or reactive arthritis. Imaging studies are also conducted to rule out osteomyelitis. After joint aspiration, the joint is immobilized for two to three days, and aggressive physical therapy is followed up to prevent muscle atrophy and restore function to the joint [2].

Differential diagnoses, associations, and comorbidities

When considering pyogenic arthritis as a diagnosis, the differential is broad. It can present with a range of mimicking agents that may present similarly with joint pain, redness, warmth, swelling, and pain on palpation or movement of the joint with a limited range of motion. Therefore, a thorough history and physical examination are crucial in elucidating the diagnosis [33]. Other causes of acute arthritis may present with a similar clinical presentation to pyogenic arthritis; therefore, arthrocentesis should be performed as soon as possible, assuming the patient is stable. Synovial fluid should be assessed for WBC count with differential, crystal analysis, gram stain, and culture to elucidate the underlying cause of arthritis. When evaluating a patient with possible pyogenic arthritis, multiple other pathologies should be considered, especially cellulitis, abscess, osteomyelitis, avascular necrosis, crystal-induced arthropathies, Lyme disease, malignancy, reactive arthritis, RA, and, in particular, transient synovitis [33]. Cellulitis most commonly presents in the lower extremities with focal areas of increased pain, warmth, erythema, swelling, and possible lymphangitis, with *Streptococcus pyogenes* and *S. aureus* being the most typical causative organisms. Diagnosis is based on clinical findings used with blood cultures, as blood cultures have a high rate of false positives. Abscess may present with a swollen, erythematous, fluctuating mass under the skin, most commonly on the legs or arms, and MRSA causes most cases. Clinical findings supplemented with ultrasound and computed tomography findings may increase the sensitivity and specificity of confirming or excluding a diagnosis of pyogenic arthritis [33].

Osteomyelitis may present with increasingly severe pain, swelling, and joint erythema. It is mainly caused by *S. aureus*, although several other bacteria are possible infectious agents. The risk of osteomyelitis may also be increased in diabetic patients, and in many cases, four or more weeks of antibiotic therapy are recommended [34]. Infection can occur in patients due to bacteremia, extension through contiguity, or direct inoculation or trauma of the affected bone. Exposed bone or a draining sinus tract may be sufficient for diagnosis, while blood cultures, C-reactive protein, and erythrocyte sedimentation rate values can also aid clinical judgment. Early infection is best identified with MRI or bone scintigraphy, while chronic infection with more advanced bone destruction may be identified on x-ray [34-37]. Avascular necrosis typically affects the acetabulum of the femur in young, healthy patients between 20 and 40 years of age. It should be suspected in the setting of femoral head or neck fractures or patients with hemoglobinopathies due to the risk of ischemia to the affected joint. Other common causes include hip dislocation, radiation, slipped-cap femoral epiphysis, myeloproliferative disorders, chronic corticosteroid use, alcohol use disorder, smoking, autoimmune disorders, and coagulopathies. Patients may present with minimal joint symptoms that may progress to a range of motion limitation at the hip with characteristic pain on internal rotation that may refer to the gluteal region or knee. In contrast to pyogenic arthritis, diagnosis is typically made via MRI in the acute setting and x-ray in the chronic setting [38-40]. In chronic cases of osteomyelitis, surgical debridement may be necessary to prevent spread or further complications. Even with surgical intervention and antibiotic therapy, recurrence rates remain high [34]. There are differences in both presentation and treatment of acute and chronic osteomyelitis between adults and children. A four-week course of antibiotics typically treats acute osteomyelitis in children, while treatment of chronic cases requires a much more extended period of antibiotic treatment. However, in both chronic and acute cases, empiric coverage of *S. aureus* is indicated [34].

Gouty arthritis is a crystal-induced arthritis typically affecting males, those with chronic alcohol intake, and increased red meat ingestion. It can closely mimic the symptoms of pyogenic arthritis; however, synovial fluid typically contains uric acid crystals, which preferentially affect the great toe joint. Calcium pyrophosphate dihydrate crystals affecting the ankle or knee joint are commonly present in pseudogout. Of note, the presence of crystals does not rule out underlying joint infection, and fluid should still be gram-stained and cultured [41,42]. Lyme disease is a tick-borne illness caused by *B. burgdorferi* with a high incidence in the Northeast and upper Midwest regions of the United States. The second stage of the disease causes migratory arthralgia that can progress to Lyme arthritis if left untreated. Lyme arthritis is typically seen during the third stage of the illness and is caused by direct inflammation of the joint tissue by *Borrelia *bacteria. The diagnosis is made clinically based on history, physical exam findings, or serologic testing with enzyme-linked immunoassay (ELISA) and western blot detecting IgG or IgM antibodies against *Borrelia*. However, if clinical signs and symptoms are present, Lyme disease should be treated empirically regardless of disease stage [43-46].

Reactive arthritis is an inflammatory arthritis associated with blood culture-confirmed infection with *Chlamydia trachomatis*, *Campylobacter jejuni*, *C. difficile*, *Escherichia coli*, *Salmonella*, *Shigella *spp., *Yersinia *spp., *Chlamydophila pneumonia*, or *Mycoplasma pneumoniae*. Infection is typically confirmed via PCR or stool culture. Synovial fluid often contains <50,000 WBC/hpf and a negative culture. Symptoms of the associated diseases in the setting of oligoarticular arthritis help identify reactive arthritis. Of note, the HLA-B27 phenotype puts patients at a significantly increased risk [47,48]. RA has the highest incidence in females aged 30-50. Patients typically experience morning stiffness for more than one hour after awakening. Distal upper extremity joints are most affected, helping to distinguish RA from pyogenic arthritis. Diagnosis may involve an extensive workup, including synovial fluid analysis, serology, and inflammatory markers. While rheumatoid factor is sensitive and may increase suspicion of the disease, diagnosis is typically confirmed with an anti-citrullinated protein antibody, which is highly specific and diagnostic [49-51]. Transient synovitis is commonly associated with preceding upper respiratory tract infection; however, the disease process is poorly understood. Patients may present with hip pain, abnormal gait, and inability to bear weight with decreased range of motion on the affected joint. Transient synovitis is a diagnosis of exclusion, and Kocher’s criteria should be utilized for risk stratification [52]. These criteria include the inability to bear weight on the affected side, erythrocyte sedimentation rate (ESR) > 40, fever > 38.5°C, and WBC > 12,000.

Lastly, ultrasound can identify effusions and aid in arthrocentesis [53]. Septic bursitis can be diagnosed based on clinical features, including significant pain, tenderness, and decreased range of motion, most commonly located in the olecranon or prepatellar bursae. Aspiration of the sac and bursal fluid analysis are considered the gold standard in diagnosis. Fluid should be assessed for cell count, Gram stain, culture, and crystals. Further, ultrasonography can help distinguish bursitis from cellulitis. Additionally, white blood cell count, inflammatory markers, and magnetic resonance imaging with increased signal intensity can aid in distinguishing infectious from noninfectious causes [54].

Clinical considerations and potential new treatment options

Prognosis

Septic arthritis is a medical emergency. Mortality rates for septic arthritis have been documented anywhere between 2% and 56% [55,56]. Even with proper treatment and antibiotic usage, there remains a 7%-15% mortality rate for patients admitted to the hospital and undergoing treatment for septic arthritis [2]. Additionally, over one-third of patients will continue to experience significant morbidity, primarily in the form of irreversible joint destruction and subsequent disability [2,55,57]. Morbidity and mortality have been shown to increase with advanced age, polyarticular involvement, presence of synthetic intra-articular materials, elevated C-reactive protein, skin involvement, bacteremia, low creatine clearance, and comorbid conditions such as pre-existing joint disease, diabetes mellitus, or rheumatoid arthritis [2,55]. Mortality is also highly dependent on the causative organism(s). Patients infected with *Staphylococcal *species have significantly increased mortality (56%) compared to infection with *Gonococcal *species, which rarely results in death [2,58].

MRSA is a particularly devastating infection as traditional antibiotic therapy may not provide sufficient infection control, leading to continued activation of host inflammatory response and cytokine release as well as ongoing joint destruction [58,59]. In most cases, oral antibiotics can be given as they are not inferior to intravenous therapy. Proper antibiotics for oral administration in the treatment of MRSA include trimethoprim/sulfamethoxazole (TMP/SMX), rifampin, linezolid, and clindamycin. Specifically, based on the source, adenosine deaminase is useful in differentiating septic arthritis from rheumatoid and crystal-induced arthritis. Higher levels of adenosine deaminase (ADA) were associated with septic arthritis (infection-related inflammation), rather than crystal-induced or osteoarthritis. The total duration of antibiotic therapy can range from two to six weeks, but certain infections may require longer courses to be completely effective [60].

Special considerations

Rheumatoid Arthritis

Patients with a history of RA have been consistently shown to have higher rates of negative outcomes associated with septic arthritis [55,61]. Patients with RA are at an enhanced risk related to compromised immune systems and the use of immunosuppressive medications [2,62,63]. These patients often lack typical clinical symptoms such as fever and leukocytosis, which may delay diagnosis and appropriate treatment [64]. Synovial fluid adenosine deaminase (ADA) has been identified as a useful marker in differentiating septic arthritis from rheumatoid and crystal-induced arthritis, aiding in accurately diagnosing septic arthritis in these patients [65]. ADA is useful in differentiating septic arthritis from rheumatoid and crystal-induced arthritis. Higher levels of ADA were associated with septic arthritis (infection-related inflammation), rather than crystal-induced or osteoarthritis. Furthermore, patients with RA often experience additional risk factors, including but not limited to prior joint destruction, history of intra-articular injections, and joint prostheses, further emphasizing the unique considerations for this population [61].

Periprosthetic Infection

Acute periprosthetic joint infection, while clinically resembling native septic arthritis, poses a unique challenge for providers, given the risk of biofilm formation [57]. Prompt recognition and treatment with open surgical debridement, antibiotics, and implant replacement within four weeks of initial arthroplasty and symptom onset are paramount in managing the disease progression [57,66]. In contrast to native septic arthritis, arthroscopic irrigation and debridement do not achieve adequate infectious source control [67]. The choice of antimicrobial and length of treatment also differs significantly for periprosthetic joint infection. Given the high rates of biofilm formation, proper antibiotics should achieve therapeutic concentrations within the bone and possess activity against the specific organisms within these biofilms [66]. Additionally, patients with suspected periprosthetic infections should receive at least three months of antimicrobial therapy compared to the standard six-week treatment in native septic arthritis [66].

Potential novel treatment

As discussed previously, a significant portion of morbidity and subsequent joint dysfunction following septic arthritis is a result of host immune response to foreign pathogens. While antibiotic treatment aims to eliminate the source of the infection and limit further immune system activation, the presence of T-cells, B-cells, and macrophages within the joint space leads to the release of an inflammatory cytokine, which ultimately results in permanent cartilage erosion and joint damage [59].

Antimicrobial peptides (AMPs), also known as host defense peptides, play a crucial role in the defense against bacterial pathogens. AMPs exhibit diverse mechanisms, including direct killing through membrane damage, chemoattraction, and regulated immune cell activation. AMPs are also shown to interfere with toll-like receptor pathways to reduce pro-inflammatory responses. AMPs also modulate macrophages and dendritic cells, preventing undue activation and suppressing the production of pro-inflammatory cytokines [68,69]. Overall, AMPs function as antibacterial peptides and contribute to immune normalization, making them ideal for managing and treating infections, especially in synergy with conventional antibiotics [70,71]. Although AMPs for the treatment of septic arthritis have not received extensive investigation, preliminary studies with recombinant C-type Lectin Domain Family 3 Member A (CLEC3A), a cartilage-specific protein, have shown significant antimicrobial activity against *E. coli*, *P. aeruginosa*, and *S. aureus*.

Similarly, titanium implants coated with CLEC3A demonstrate reduced bacterial adhesion, indicating some potential for use in periprosthetic joint infections [72]. Other AMPs, such as LyeTxI-b, a synthetic peptide derived from *Lycosa erythrognatha *spider venom, have been shown to significantly reduce the bacteria burden and the number of inflammatory cells within the joint. Adding clindamycin with LyeTxI-b showed an even more significant reduction in inflammatory cytokines, indicating a possible synergistic relationship between AMPs and antibiotics traditionally used in treating septic arthritis [73]. Elevated matrix metalloproteinases (MMPs) in septic arthritis often exacerbate joint destruction through extracellular matrix degradation, leading to irreversible cartilage damage [74,75]. Animal models with deficient MMP-7 showed significantly less cartilage destruction both clinically and histologically when compared to wild type following inoculation with *S. aureus* [76]. Similarly, newer studies have shown that inactivation of MMP-2 results in significantly less swelling, reduced bacterial load, and reduced inflammatory cytokines [77]. These studies indicate a possible role for MMP neutralization in reducing joint destruction associated with septic arthritis.

In addition to the use of AMPs and MMP neutralization, animal models have demonstrated that blockade of various interleukins, including IL-33, IL-15, and IL-4, may help clear bacterial infections while also limiting cytokine-induced joint destruction [78-80]. Conversely, IL-10 appears to have a protective effect, whereas mice with a deficient IL-10 gene had a higher bacteria burden and greater joint destruction [81,82].

## Conclusions

This investigation explored pyogenic arthritis and focused on various intricacies and relationships between causative pathogens and joint environment. As a relatively uncommon but essential affliction, pyogenic arthritis poses significant challenges to both patients and healthcare providers due to its complicated profile. Establishing a correct diagnosis via positive culture and clinical symptoms is essential in administering the proper course of treatment while also considering patient-specific factors such as allergies to different medications. It is necessary to facilitate a cooperative working environment between physicians, nurses, support staff, and patients to execute an effective treatment plan while ensuring positive outcomes and preventing negative ones. Providers must exercise compassion while acquiring an adequate history and understanding possible risk factors predisposing the patient to develop pyogenic arthritis. Once the diagnosis has been established, reducing mortality and suffering is of utmost importance. Patients achieve better survival rates and outcomes with prompt action and effective treatment.
